# Forward and Backward Private Searchable Encryption for Cloud-Assisted Industrial IoT

**DOI:** 10.3390/s24237597

**Published:** 2024-11-28

**Authors:** Tianqi Peng, Bei Gong, Shanshan Tu, Abdallah Namoun, Sami Alshmrany, Muhammad Waqas, Hisham Alasmary, Sheng Chen

**Affiliations:** 1 College of Computer Science, Beijing University of Technology, Beijing 100124, China; tianqi_peng@emails.bjut.edu.cn (T.P.); gongbei@bjut.edu.cn (B.G.); sstu@bjut.edu.cn (S.T.); 2 AI Center, Faculty of Computer and Information Systems, Islamic University of Madinah, Madinah 42351, Saudi Arabia; a.namoun@iu.edu.sa (A.N.); s.alshmrany@iu.edu.sa (S.A.); 3 School of Computing and Mathematical Sciences, Faculty of Engineering and Science, University of Greenwich, London SE10 9LS, UK; 4 School of Engineering, Edith Cowan University, Joondalup, Perth, WA 6027, Australia; 5 Department of Computer Science, College of Computer Science, King Khalid University, Abha 61421, Saudi Arabia; alasmary@kku.edu.sa; 6 School of Electronics and Computer Science, University of Southampton, Southampton SO17 1BJ, UK; sqc@soton.ac.uk; 7 Faculty of Information Science and Engineering, Ocean University of China, Qingdao 266005, China

**Keywords:** cloud-assisted IIoT, symmetric searchable encryption, forward and backward privacy, state chain structure

## Abstract

In the cloud-assisted industrial Internet of Things (IIoT), since the cloud server is not always trusted, the leakage of data privacy becomes a critical problem. Dynamic symmetric searchable encryption (DSSE) allows for the secure retrieval of outsourced data stored on cloud servers while ensuring data privacy. Forward privacy and backward privacy are necessary security requirements for DSSE. However, most existing schemes either trade the server’s large storage overhead for forward privacy or trade efficiency/overhead for weak backward privacy. These schemes cannot fully meet the security requirements of cloud-assisted IIoT systems. We propose a fast and firmly secure SSE scheme called Veruna to address these limitations. To this end, we design a new state chain structure, which can not only ensure forward privacy with less storage overhead of the server but also achieve strong backward privacy with only a few cryptographic operations in the server. Security analysis proves that our scheme possesses forward privacy and Type-II backward privacy. Compared with many state-of-the-art schemes, our scheme has an advantage in search and update performance. The high efficiency and robust security make Veruna an ideal scheme for deployment in cloud-assisted IIoT systems.

## 1. Introduction

As an extension of the Internet of Things (IoT) to the industrial field, the industrial IoT (IIoT) plays a crucial part in Industry 4.0 and smart cities [1,2,3]. Massive industrial data are monitored, collected, and analyzed by physical devices deployed in IIoT, such as IoT nodes, sensors, and actuators, which promote productivity, efficiency, safety, and other economic benefits [4,5]. In addition, with the rapid development of cloud computing, the cloud-assisted IIoT system, which outsources industrial data to the cloud server, can provide more robust data processing and flexible storage space. However, since the cloud server may not always be trusted, data privacy leakage becomes a critical consideration. Intuitively, this problem can be solved by encrypting data and uploading it to the server. Since encrypted data lose their processing flexibility and cannot be used to retrieve data, researching how to perform effective retrieval on ciphertext becomes a new challenge.

Symmetric searchable encryption (SSE) was proposed to address the above issue. SSE is designed on symmetric cryptographic primitives, achieving ciphertext retrieval by generating an encrypted index for each encrypted file. The encrypted files and corresponding indexes are generally uploaded to the server. When retrieving the files containing a specific keyword, the client can generate a search token that encrypts the keyword and sends the search token to the cloud server. Then, the server uses the search token and the encrypted index to execute the search algorithm and returns the relevant file to the client [6].

Although the early SSE scheme based on static data allows ciphertext retrieval [7,8,9], it needs to leak some information to the server to achieve this function. This leakage includes search patterns and access patterns. The search pattern reveals which queries correspond to the exact keywords, and the access pattern reveals which files were received by a search query [10]. It is worth noting that the static-based scheme makes it difficult to achieve the dynamic data update in IIoT. To support adding or deleting files, Kamara et al. [11] introduced the dynamic SSE (DSSE). However, DSSE needs to leak additional privacy to trade for updates. These leakages include the following two cases: (a) when inserting a file into the database, it will reveal whether the file contains a keyword that has been searched before, and (b) when searching for a specific keyword, the files that contain the keyword but have been deleted from the database will still be retrieved. To avoid the leakage of the above private information and implement the DSSE more securely in IIoT, it is necessary to introduce forward and backward privacy [12].

The notion of forward privacy and backward privacy was introduced by Stefanov et al. [13]. Forward privacy ensures that the newly inserted files cannot be linked with the previous search queries. DSSE schemes with forward privacy can resist powerful file injection attacks, which exploit the leakage in case (a). Backward privacy ensures that the current search query cannot be linked with the deleted files, which is leakage in case (b). Therefore, when a search query on keyword *w* is issued, the DSSE scheme with backward privacy does not retrieve the deleted files containing *w*.

The forward-private DSSE schemes [14,15,16,17,18,19,20,21] offer some original effective results. However, the structures in these schemes need more consideration of backward privacy, and they require a large storage overhead on the server side, which will reduce the efficiency of data processing. The DSSE with backward privacy is comparatively more recent. Bost et al. [22] formally defined three types of backward privacy. Specifically, **Type-I** reveals the number of updates (insertions and deletions) on keyword *w*, the current file identifiers containing *w*, and their insertion timestamps. **Type-II** additionally leaks the timestamp of each update for *w*, while **Type-III** further reveals precisely which delete operation cancels which insert operation. The security progressively weakens from **Type-I** to **Type-III**. Subsequently, a few schemes with backward privacy [23,24,25,26,27,28] have been proposed recently. These schemes introduce additional cryptographic primitives (e.g., Homomorphic Encryption (HE), Puncturable Encryption (PE), Symmetric Revocable Encryption (SRE), etc.) to support backward privacy. However, these cryptographic primitives undoubtedly increase the cryptographic operations in the server and search overhead. More importantly, most of these schemes only achieve **Type-III** backward privacy. Note that **Type-III** backward privacy leaked the timestamp of when the files were deleted. For IIoT systems, time is critical information that many attacks [29,30] can exploit to break the system’s security. For DSSE, the adversary can correlate the information of subsequent queries or make statistical inferences based on when the file was deleted. Therefore, the scheme with **Type-III** backward privacy cannot meet the systems’ security requirements.

To sum up, most existing schemes either trade the server’s large storage overhead for forward privacy or trade efficiency/overhead for Type-III weak backward privacy.

### Our Contributions

To address the above issues, we are facing the following two challenges, more specifically, (1) how to design a practical structure that requires less server storage while maintaining the property of forward privacy, and (2) how to achieve **Type-II** backward privacy without compromising search efficiency. This paper addresses these challenges and presents Veruna, which is an efficient DSSE scheme with strong forward and backward privacy.

We first design a novel state chain structure that shares a similar basic idea as the keyed-based blocks chain (KBBC) structure in [17], encrypting file identifiers with random keys stored in the client side to achieve forward privacy. However, our state chain structure uses a state token to track the state of each keyword instead of storing additional blocks of file identifiers, as in KBBC, which reduces the storage overhead. For backward privacy, the key to achieving Type-II backward privacy on our state chain structure without compromising search efficiency is achieved by reducing leakage during the search with fewer cryptographic operations. To this end, we implement a simple yet effective approach to accomplish deletions locally with simple cryptographic operations in the server while capturing the essential properties of Type-II backward privacy. Veruna’s key contributions are summarized below. We also summarize the comparison of our scheme, Veruna, with other existing DSSE schemes in Table 1.

- We design a novel state chain structure that links the keyword’s state nodes for each update through a state token. As each state of the keyword is randomly generated and the state token is only associated with the current and previous states, the previous search queries cannot be linked to the future state of the keyword. Therefore, our scheme achieves the property of forward privacy. In addition, less storage space is required in the server to support the state chain structure for executing search operations.
- To achieve **Type-II** backward privacy without compromising search efficiency, we reduce leakage during the search using a simple local deletion method. The scheme directly sends encrypted entries retrieved by the server to the client during the search. The client decrypts them locally and filters out the deleted files. This method ensures the server cannot correlate current search queries with deleted files while only revealing the update timestamp during the search. The cryptographic operations involved are simple XOR operations, which enhance search efficiency and make the scheme practical for IIoT systems.
- We analyze the security of our scheme and show that it features forward privacy and **Type-II** backward privacy. We also compared the proposed scheme with state-of-the-art schemes through experiments to evaluate its performance.

The organization of this paper is as follows. Section 2 reviews the related work, and Section 3 introduces the cryptographic background and relevant definitions of DSSE used in this paper. The proposed scheme and its security analysis are presented in Section 4. The performance evaluation of the scheme can be found in Section 5. Finally, Section 6 provides the conclusion and future work.

## 2. Review of Existing Works

The first SSE scheme was introduced by Song et al. [7], which was based on a linear search time construction. This led to some further work on SSE. However, all these works focused on static settings, which cannot achieve the real-time update of large data in IIoT. In 2012, Karama et al. [11] formally introduced the notion of DSSE and proposed a DSSE scheme, which allows the client to add or delete data from the database. Subsequently, a series of works were carried out focusing on DSSE function [31,32,33], security [34,35,36], and efficiency [35,37,38]. In particular, due to the rich expressiveness of attribute-based encryption (ABE), Liu et al. [39] and Yu et al. [40] used attribute-based encryption and blockchain technology to implement fine-grained search and revocable functions. Yin et al. [41] designed a novel access policy-based secure index and an attribute-based search token, which enable the scheme to achieve a fine-grained search while implementing access control. Li et al. [42] introduces an efficient electronic medical records (EMR) management model OLOS and a quantum-resistant KS-ABE scheme, enhancing data security, reducing communication costs, and ensuring secure cross-institutional EMR sharing. Although they have effectively reduced the overhead in decryption and revocation processes, DSSE schemes based on ABE are still challenging to apply in resource-constrained network environments. Moreover, some works focused on the common query and result pattern leakage in SSE protocols. Yang et al. [43] developed OpenSE, which is a verifiable searchable encryption scheme utilizing the oblivious polynomial evaluation (OPE) protocol to protect query and result pattern privacy. Xu et al. [44] addressed keyword pair result pattern (KPRP) leakage by proposing a DSSE scheme to counter this vulnerability. Chen et al. [45] introduced MFSSE, which was an SSE scheme that hides search patterns by altering the search trapdoor for each query and incorporating random errors to defend against access pattern leakage.

Forward and backward privacy can effectively control the leakage in DSSE. The notion of forward privacy was first proposed by Chang and Mitzenmacher [46] in 2005. Several DSSE schemes were initially based on the oblivious RAM (ORAM) structure to achieve forward privacy, but they suffer from high communication costs. In 2016, Bost [14] proposed a seminal forward private scheme called Sophos, which uses a one-way trapdoor permutation to achieve forward privacy with a low communication cost. However, the performance bottleneck of Sophos is based on public key cryptographic primitives. Subsequently, many works [17,18] further optimized this scheme. In particular, Wei et al. [17] designed key blockchain structures based on symmetric cryptographic primitives to achieve forward privacy. Guo et al. [47] proposed a dual indexing structure to achieve conjunctive keyword search with forward privacy. Li et al. [48] proposed a forward privacy scheme for healthcare systems using a triple dictionary structure, but complex update operations hinder the scheme’s efficiency. Wang et al. [20] established a trapdoor permutation function based on symmetric cryptographic primitives to ensure forward privacy in their scheme. However, only considering forward privacy and neglecting backward privacy can make applying a DSSE scheme in practice challenging.

Several schemes with backward privacy were recently proposed [22,23,24,25,26,27,28,49]. To be more specific, Bost et al. [22] formally defined backward privacy and proposed several schemes with different leakages. Their first scheme, called Fides, is a **Type-II** construction, while their other two schemes, referred to as Diana and Janus, are both **Type-III** constructions based on puncturable encryption (PE). The fourth scheme of [22], known as Moneta, achieves **Type-I** backward privacy based on the ORAM [19]. Later, Sun et al. [23] pointed out that the PE in Janus is a public key cryptographic primitive, and its deletion efficiency is low. Therefore, the authors of [23] constructed a symmetric PE primitive using the pseudo-random function to improve the update efficiency. However, this scheme only achieves backwards privacy in **Type-III**. At the same time, Chamani et al. [24] proposed three improved schemes: Mitra, Orion, and Hours. Mitra, a **Type-II** scheme, performs better than Fides [22] by using symmetric key encryption. Orion is a **Type-I** scheme based on ORAM, and Hours, a **Type-III** scheme, optimizes the performance of Orion at the cost of leaking more information. Demertzis et al. [26] proposed a QoS scheme to reduce client-side storage. To the best of our knowledge, Qos is the first quasi-optimal **Type-III** backward privacy scheme. Sun et al. [27] first introduced a new symmetric revocable encryption (SRE) in the DSSE scheme. All these schemes make different trade-offs between security and efficiency. However, most existing schemes rely on additional cryptographic primitives or achieve only weak **Type-III** backward privacy. It should also be noted that although a few schemes, such as Moneta [22] and Orion [24], achieve **Type-I** backward privacy, their huge communication and computation overhead limit their potential for adoption in practice [22,24,26,27,50]. Also, Orion incurs many rounds of client–server communication during searches.

In addition, some hardware-based schemes [28,49] achieved different types of backward privacy. For example, Amjad et al. [49] used Intel SGX [51] to propose several backward-private schemes. Unfortunately, some works [30,52,53] have identified security vulnerabilities in SGX, which presents a potential risk to the schemes of [49]. More recently, some state-of-the-art schemes focus on innovations in the expressive power of DSSE. Chen et al. [54] employed the idea of inner product matching to realize conjunctive keyword search and introduced a mechanism for duplicate data deletion. Li et al. [55] proposed a scheme with verifiable functionality for Boolean keyword queries using puncturable encryption. Chen et al. [56] proposed a searchable encryption scheme with verification capability for medical data using blockchain and hash-proof chain.

In summary, more research is still needed on the security and efficiency of DSSE.

## 3. Preliminaries

In this section, we introduce the notions used in this paper, the cryptographic background, and related definitions involved in dynamic searchable encryption.

### 3.1. Notations

We use $x\overset{\$}{\to }X$ to denote that *x* is uniformly and randomly sampled from the finite set *X*. Given a sufficiently large security parameter λ∈N, a function μ:N→R is said to be a negligible function in λ if for each positive polynomial *p*, $\mu \left(\lambda \right)<\frac{1}{p(\lambda )}$ always holds. We denote by poly(λ) and negl(λ) unspecified polynomial and negligible functions in λ, respectively.

We store all documents containing keyword w∈W in the database DB as keyword-file identifier pair (w,ind), where ind is the file identifier, and W denotes the set of all keywords that appear in DB. We denote by |W| the number of distinct keywords and by DB(w) the set of file identifiers that contain the keyword *w*.

### 3.2. Searchable Encryption

A DSSE scheme consists of an algorithm Setup and two protocols Update and Search run by the client and the server.

Setup(λ,DB) is an algorithm that generates keys and constructs an encrypted database. It takes the security parameters and the database as inputs and outputs (sk,σ;EDB), where sk is the client’s secret key, σ is the local state for the client, and EDB is an encrypted database, which is initially empty.

Update(sk,σ,w,ind,op;EDB) is a client–server protocol for adding an entry to or removing an entry from a database. The client takes the secret key sk, the local state σ, an operation type op, and a keyword-file identifier pair (w,ind) as inputs. The server takes the encrypted database EDB as input. The protocol outputs the updated local state to the client and the updated encrypted database to the server as requested by the client.

Search(sk,w,σ;EDB) is a client–server protocol for searching the database corresponding to keyword *w*. The client’s inputs include the secret key sk, the keyword *w*, and the local state σ. The server’s input is the encrypted database EDB. The protocol generates DB(w) as output, and the client’s local state σ and the encrypted database EDB may also be modified.

The above contents follow the formal definition of dynamic searchable encryption in [24]. However, other works [15,16] use different definitions for dynamic searchable encryption that takes as input an entire file for addition/deletion in the Update protocol, and the protocol adds/removes all the relevant keywords to/from the database. This is functionally equivalent to executing the multiple above Update protocol on the relevant keyword-identifier pairs. Finally, we implicitly assume that after receiving DB(w), the client still needs additional interaction with the server to obtain the actual files.

### 3.3. Definitions of Correctness and Security

**Correctness**. The correctness of a dynamic searchable encryption scheme Σ=(Setup,Update,Search) means that for each query *q* and database DB, the search protocol always returns the correct result DB(q).

**Security**. Informally, an SSE scheme is secure if no more information is leaked than allowed. The security of searchable encryption was first formalized by Curtmola et al. [8]. Specifically, through two games $R_{\mathrm{EAL}}$ and $I_{DEAL}$, a secure scheme with leakage function L should reveal nothing other than this leakage. The leakage function $L=(L^{Stp},L^{Updt},L^{Srch})$ is used to capture all information learned by the adversary, where $L^{Stp}$, $L^{Updt}$ and $L^{Srch}$ denote the information leaked by Setup, Update and Search, respectively.

**Definition** **1** (Adaptive Security of DSSE)**.** *Let Σ=(Setup,Update,Search) be a dynamic searchable encryption scheme, A be a probabilistic polynomial-time (PPT) adversary, and S be a simulator. The games ${R_{EAL}}_{A}^{\Sigma }\left(\lambda \right)$ and ${I_{DEAL}}_{A,S}^{\Sigma }\left(\lambda \right)$ are defined as follows.*

- *${R_{EAL}}_{A}^{\Sigma }\left(\lambda \right)$: Adversary A chooses an initial database DB, and this game returns the encrypted database EDB to A by running Setup(˘,DB). Then, A adaptively performs a series of queries containing both search and update queries. For a search query, the game runs Search(sk,w,σ;EDB), and for an update query, the game runs Update(sk,σ,ind,w,op;EDB). Finally, Adversary A observes real results of all operations and outputs a bit b∈{0,1}.*
- *${I_{DEAL}}_{A,S}^{\Sigma }\left(\lambda \right)$: Adversary A chooses an initial database DB. Simulator S uses the leakage function $L^{Stp}\left(\mathrm{DB}\right)$ to generate an encrypted database $\mathrm{EDB}\leftarrow S(L^{Stp}\left(\mathrm{DB}\right))$ and returns it to A. Then, A adaptively performs a series of queries. For a search query q, Simulator S runs $S(L^{Srch}\left(q\right))$, and for an update query q, S runs $S(L^{Updt}\left(q\right))$. Finally, Adversary A observes the simulated results of all operations and outputs a bit b∈{0,1}.*

*The dynamic searchable encryption scheme* Σ *is said to be L-adaptively-secure if for all PPT adversaries A, there exists a simulator S in the above game model such that*

(1) $$|\mathrm{Pr}\left({R_{\mathrm{EAL}}}_{A}^{\Sigma }\left(\lambda \right)=1\right)-\mathrm{Pr}\left({I_{\mathrm{DEAL}}}_{A,S}^{\Sigma }\left(\lambda \right)=1\right)|\leq negl\left(\lambda \right).$$

### 3.4. Forward and Backward Privacy

A dynamic, searchable encryption scheme can achieve the real-time update of data, including adding and deleting files. However, more information will be leaked during the update. Achieving forward and backward privacy for dynamic searchable encryption can control the information leakage in the update operation [13,22].

**Forward privacy**. This security property is focused on file addition operations to ensure that an update query leaks no information about the keywords to be updated. A searchable encryption scheme without forwarding privacy leaks information when inserting a file. Based on this information, the server tests the previous search token on the new update to observe whether the old search token matches the new update. A file-injection attack exploits this point to recover information about the keyword. First, the server can trick the client into injecting some files containing certain specific keywords. After the client has uploaded the injected files, the server uses the search token previously submitted by the client to search for the injected files and recovers the keyword corresponding to the token based on the search result. This attack can be prevented, since forward privacy ensures the previous search token cannot match a newly inserted file.

**Definition** **2** (Forward Privacy [17])**.** *An L-adaptively-secure SSE scheme with forward privacy ensures that for an update query $q_{i}=\left(w_{i},ind_{i},op\right)$, the leakage function is $L^{Updt}\left(q_{i}\right)=\left(ind_{i},op\right)$ during the update.*

**Backward privacy**. This security property focuses on file deletion operations to ensure that when a keyword-identifier pair (w,ind) is inserted into and removed from the database, subsequent search queries on *w* do not reveal the identifier ind. A searchable encryption scheme with backward privacy should leak nothing about the deleted file’s identifier. Formally, the definition of backward privacy was given in [22], which includes three different types of backward privacy, namely, **Type-I**, **Type-II** and **Type-III**, and from **Type-I** to **Type-III**, the security becomes progressively weaker. Before going into the details of these definitions, we refer to the notions in [22] to introduce some functions involved in backward security.

The leakage function holds a list of all queries Q. The search query is stored in the entry as (u,w), and the update query is stored in the entry as (u,op,(ind,w)), where *u* is the timestamp of the query, and op=add/del. For keyword *w*, search pattern sp(w) records the timestamps of all search queries on *w*, which are formally defined as

(2) sp(w)={u|(u,w)∈Q}.

TimeDB(w) is a list of the files that contain keyword *w*, but the list does not contain information about the files that have been removed from the database DB. Formally, it is defined as

(3) $$\begin{array}{cc} \mathrm{TimeDB}(w)= & \{(u,ind)|(u,add,(w,ind))\in Q\\ \; & \mathrm{and}\;\forall u^{\prime },\left(u^{\prime },del,\left(w,ind\right)\right)\notin Q\}. \end{array}$$

Note that each entry in TimeDB(w) is stored as a timestamp-identifier pair (u,ind). Updates(w) is a list of update timestamps for keyword *w*, which is formally defined as

(4) $$\begin{array}{cc} \mathrm{Updates}(w)= & \{u|(u,add,(w,ind))\in Q\\ \; & \mathrm{or}\;(u,del,(w,ind))\in Q\}. \end{array}$$

The timestamp of insertion and deletion of keyword-identifiers pair (w,ind) is contained in Updates(w). Finally, DelHist(w) is a list of the history of deleted entries for keyword *w*. Formally, DelHist(w) is defined as

(5) $$\begin{array}{cc} \; & \mathrm{DelHist}\left(w\right)=\{\left(u^{add},u^{del}\right)|\;\exists \;ind\;\;s.t.\;(u^{del},del,\\ \; & \;\left(w,ind\right))\in Q\;\mathrm{and}\;\left(u^{add},add,\left(w,ind\right)\right)\in Q\}. \end{array}$$

Using the above functions, we refer to the definitions of backward privacy in [22,23,24] and make minor modifications. In [22,23,24], the definition of backward privacy does not explicitly include the common leakage sp(w) in the search protocol. However, this leakage has to be considered in these schemes. We take sp(w) into account and define the notion of backward privacy as follows.

**Definition** **3** (Backward Privacy)**.** *An L-adaptively-secure SSE scheme has backward privacy of*

(6) $$\begin{array}{cc} \mathrm{Type}\text{-}I: & \;\mathrm{if}\;L^{Updt}\left(op,w,ind\right)=L^{\prime }\left(op\right)\;\mathrm{and}\;\\ \; & \;L^{Srch}\left(w\right)=L^{\prime \prime }\left(\mathrm{sp}\left(w\right),\mathrm{TimeDB}\left(w\right),a_{w}\right), \end{array}$$

(7) $$\begin{array}{cc} \mathrm{Type}\text{-}\mathrm{II}: & \;\mathrm{if}\;L^{Updt}\left(op,w,ind\right)=L^{\prime }\left(op,w\right)\;\mathrm{and}\;\\ \; & \;L^{Srch}\left(w\right)=L^{\prime \prime }\left(\mathrm{sp}(w),\mathrm{TimeDB}(w),\mathrm{Updates}(w)\right), \end{array}$$

(8) $$\begin{array}{cc} \mathrm{Type}\text{-}\mathrm{III}: & \;\mathrm{if}\;L^{Updt}\left(op,w,ind\right)=L^{\prime }\left(op,w\right)\;\mathrm{and}\\ \; & \;L^{Srch}\left(w\right)=L^{\prime \prime }\left(\mathrm{sp}(w),\mathrm{TimeDB}(w),\mathrm{DelHist}(w)\right). \end{array}$$

*For keyword w, **Type-I** reveals the number of updates (insertions and deletions) associated with w, the file identifiers currently containing w, and when they were inserted into the database. Apart from the leakage in **Type-I**, **Type-II** also leaks each update’s timestamp and operation type involving w. **Type-III** further leaks which deletion operation cancelled which insertion operation in addition to the leakage in **Type-II**. It can be seen that from **Type-I** to **Type-III**, the security is progressively weaker.*

## 4. Veruna: Fast and Strong Security Symmetric Searchable Encryption Scheme

This section presents Veruna, which is our searchable encryption scheme with forward and backward privacy. We design a new state chain structure and a simple yet effective approach to ensure that nothing about the encrypted entry is leaked to the server during the updates, and only the timestamp of the updated entry is leaked during the searches. Compared to the existing schemes [17,22,23], our construction achieves a stronger **Type-II** backward privacy without relying on additional techniques while reducing server storage space.

### 4.1. System Model

**Figure 1:**
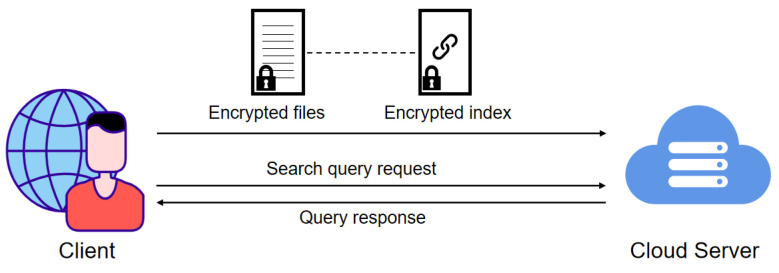
The system model of Veruna.

The system model of our scheme is shown in Figure 1, which contains two entities: the client and cloud server, as detailed below.

**Figure 2:**
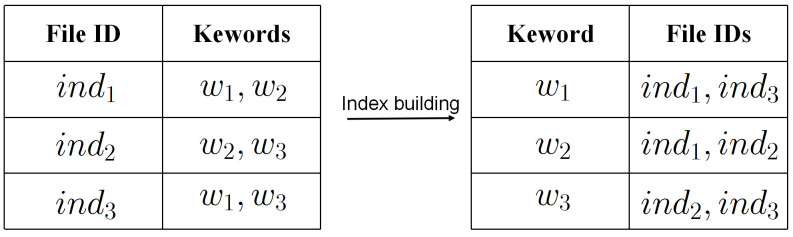
Secure index database building.

Client: The client is responsible for encrypting files and constructing secure indexes based on the keywords contained in the files. As shown in Figure 2, the client constructs an inverted index for the files in the database, where each entry in the indexed database corresponds to a keyword and the file identifiers containing that keyword. Subsequently, the client sends the encrypted files and corresponding encrypted secure indexes to the cloud server. In addition, the client can either be the data owner or the data user. When clients query the file containing a specific keyword, they must generate a search query request based on the keyword and send it to the cloud server. In this paper, we default to the client being legal.

Cloud Server: The cloud server is responsible for storing encrypted files and indexes and executing search algorithms based on the client’s search requests. Specifically, when the server receives a keyword query request from a client, it retrieves all file identifiers that meet the criteria and returns them to the client. In addition, we assume the server is honest but curious. It honestly executes update and search protocols and returns the correct search results to the client. However, the cloud server may extract privacy information from the client’s data.

In practical IoT applications, the system model could manage and query encrypted sensor data. For instance, consider a healthcare IoT system where wearable medical devices continuously monitor patient vitals such as heart rate, blood pressure, and temperature. The data collected by these devices are encrypted and stored on a cloud server. Each set of sensor readings represents a file and the associated metadata (e.g., patient ID, timestamp, and type of reading) form keywords for search queries. Using this DSSE system model, authorized healthcare providers could securely search the cloud storage using encrypted keywords to retrieve relevant files, such as all heart rate files. This process ensures the confidentiality of patient data and allows efficient data retrieval.

### 4.2. Our Construction

Wei et al. [17] proposed a forward privacy scheme whose index structure consists of keyed-block chains. Although this structure does not consider the backward privacy and its large server storage overhead, the idea of the state chain structure is attractive. Further inspired by the hash linked list, we use state tokens to establish a state chain structure based on symmetric cryptography primitive, improving forward privacy efficiency and achieving Type-II backward privacy by a simple yet effective approach. Specifically, the client randomly generates the current state every time and links the current state to the previous state by a state token. Then, the server uses a key-value dictionary that stores state tokens and encrypted index entries. During the search, the server uses the search token sent by the client to generate the locations and obtains the current state token and the corresponding encrypted index entry accordingly. However, there is no decryption operation on the encrypted index entry in the server. Since the server cannot infer the future state based on the currently known states and state tokens, and the size of each search token is fixed, our scheme achieves forward privacy. Veruna further achieves backward privacy because the server uses the search token to generate the locations, but the encrypted entry’s decryption happens locally at the client.

**Figure 3:**
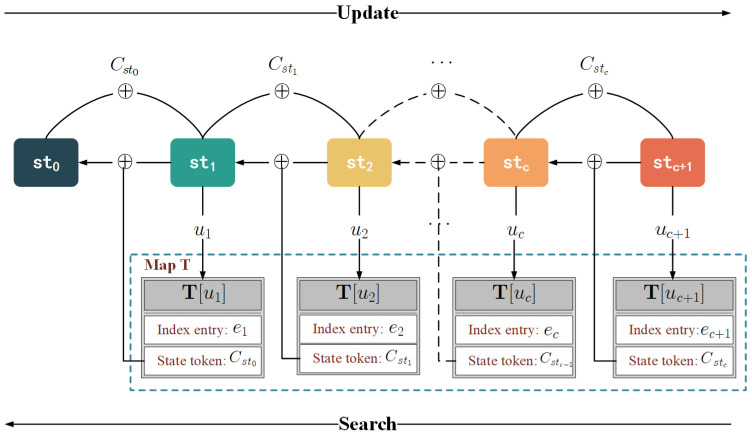
The structure of state chain.

Algorithm 1 summarizes the setup, update and search protocols of Veruna. Specifically, the structure of state chains is shown in Figure 3, while **Setup**, **Update**, and **Search** protocols are detailed below.

**Setup**. The client generates a secret key $k_{s}$ and an empty map W in the setup protocol. The key $k_{s}$ is constructed from a λ-bits random string that is used to encrypt keywords, and the map W is used to store the state of each keyword. The server also generates an empty map T, which is used to store the encrypted index.

**Update**. In the update procedure, when updating (op=add/del) a file that contains the keyword *w* and whose identifier is ind, the client first needs to obtain some variables from map W, containing the previous state of the keyword *w* and a counter *c* that denotes the update times of the keyword *w*. Then, it generates a random current state, and map W is updated (lines 5–10). Next, the client runs the hash functions $H_{1}\left(k_{w},st_{c+1}\right)$, $H_{2}\left(k_{w}^{*},c+1\right)$ and $H_{3}\left(k_{w},st_{c+1}\right)$ with key $k_{w}$ or $k_{w}^{*}$, respectively. $H_{1}\left(k_{w},st_{c+1}\right)$ is used as location *u* to indicate which encrypted index entry *e* is stored in the server; $H_{2}\left(k_{w}^{*},c+1\right)$ is used to encrypt (ind||op) and output the encrypted index entry *e*. In comparison, the $H_{3}\left(k_{w},st_{c+1}\right)$ output is XORed with the previous state, and the result becomes the state token which evolves the state (lines 11–13). Finally, the client sends $\left(u_{c+1},\left(e_{c+1},C_{st_{c}}\right)\right)$ to the server who stores it as $T\left[u_{c+1}\right]\;=\;\left(e_{c+1},C_{st_{c}}\right)$.
**Algorithm 1.** Veruna with forward and backward privacy$\underline{\mathrm{Setup}(\lambda )}$13: $C_{st_{c}}\leftarrow st_{c}⊕H_{3}\left(k_{w},st_{c+1}\right)$26: $\;\mathrm{Val}\left[i\right]\leftarrow e_{i}$Client:14: $\mathrm{Send}\;(u_{c+1},e_{c+1},C_{st_{c}})\;\mathrm{to}\;\mathrm{server}$27: $\;st_{i-1}\leftarrow C_{st_{i-1}}⊕H_{3}\left(k_{w},st_{i}\right)$1: $k_{s}\overset{\$}{\leftarrow }{\left\{0,1\right\}}^{\lambda }$Server:28: endfor2: W←emptymap15: $T\left[u_{c+1}\right]\;=\;\left(e_{c+1},C_{st_{c}}\right)$29: SendValtoclientServer:$\underline{\mathrm{Search}(k_{s},W,w;T)}$Client:3: T←emptymapClient:30: Res←∅$\underline{\mathrm{Update}(k_{s},W,w,ind,op;T)}$16: $k_{w}\;\left|\right|\;k_{w}^{*}\leftarrow F(k_{s},w)$31: fori=1to|Val.size|doClient:17: $\left(st_{c},c\right)\leftarrow W\left[w\right]$32: $\;(ind_{i}||op_{i})\leftarrow \mathrm{Val}\left[i\right]⊕H_{2}\left(k_{w}^{*},i\right)$4: $k_{w}\;\left|\right|\;k_{w}^{*}\leftarrow F(k_{s},w)$18: $\mathrm{if}\;(st_{c},c)\;=\;⊥\mathrm{then}$33: $\;\mathrm{if}\;op_{i}\;=\;add\;\mathrm{then}$5: $\left(st_{c},c\right)\leftarrow W\left[w\right]$19:return∅34: $\;\;\mathrm{Res}\leftarrow \mathrm{Res}\cup ind_{i}$6: $\mathrm{if}\;(st_{c},c)\;=\;⊥\mathrm{then}$20: endif35: else7: $\;{st}_{0}\overset{\$}{\leftarrow }{\left\{0,1\right\}}^{\lambda }\;,\;c\leftarrow 0$21: $\mathrm{Send}\;(k_{w},st_{c},c)\;\mathrm{to}\;\mathrm{server}$36: $\;\;\mathrm{Res}\leftarrow \mathrm{Res}\setminus ind_{i}$8: endifServer:37: endif9: ${st}_{c+1}\overset{\$}{\leftarrow }{\left\{0,1\right\}}^{\lambda }$22: Val←∅38: endfor10: $W\left[w\right]\leftarrow \left(st_{c+1},c+1\right)$23: fori=cto1do39: ReturnRes11: $u_{c+1}\leftarrow H_{1}\left(k_{w},st_{c+1}\right)$24: $\;u_{i}\leftarrow H_{1}\left(k_{w},st_{i}\right)$
12: $e_{c+1}\leftarrow \left(ind||op\right)⊕H_{2}\left(k_{w}^{*},c+1\right)$25: $\;\left(e_{i},C_{st_{i-1}}\right)\leftarrow T\left[u_{i}\right]$


**Search**. To search all files containing keyword *w*, the client first runs a pseudo-random function *F* with the key $k_{s}$ to encrypt the keyword *w* and retrieves the current state and counter *c* from W[w] (lines 16–20). Then, the client sends the search token that contains the encrypted keyword, current state, and counter to the server (line 21). Given the search token, the server can compute the location of the current state and retrieve the corresponding encrypted index entry and state token from map T. The encrypted index entry is then stored in the list Val, and the server uses the state token and the current state to infer a previous state (lines 24–27). Iteratively, the server obtains all states and corresponding encrypted index entries about keyword *w* and sends the list Val containing all the encrypted index entries to the client. Upon receiving these encrypted values, the client decrypts them to obtain (ind||op) (line 32). If op=add, the corresponding ind is stored in the list Res; otherwise, the corresponding ind is removed from the list Res (lines 33–36).

**Figure 4:**
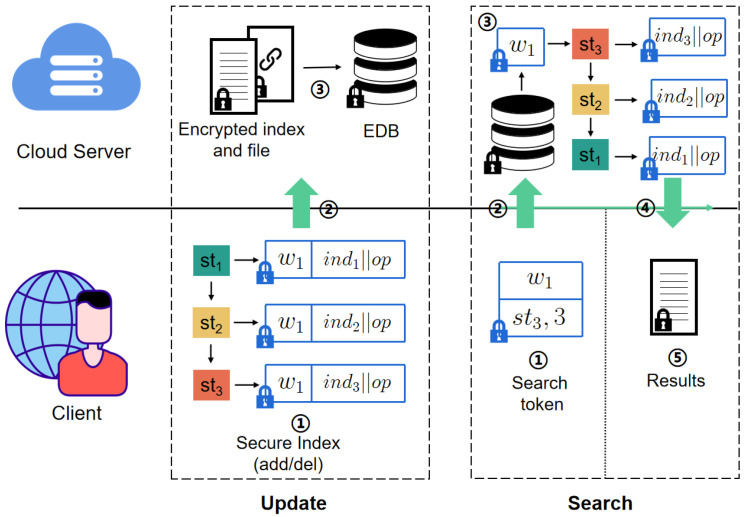
The flowchart of Veruna.

The above content presents the execution details of each protocol. For easier comprehension, Figure 4 with a brief introduction given in Table 2 provides a higher-level perspective, illustrating the interaction flow between the client and the cloud server during the update and search. In brief, if updating keyword/identifier pairs $\left(w_{1},ind_{1}\right)$, $\left(w_{1},ind_{2}\right)$, and $\left(w_{1},ind_{3}\right)$ sequentially, the client first generates a random state $st_{i},i=1,2,3$ for each pair to be updated. Then, these states are connected to construct the secure index, and the encrypted file and index are sent to the server. Upon receiving the information, the server stores the encrypted files and their corresponding secure index in the encrypted database EDB. To search for files containing the keyword $w_{1}$, the client generates relevant search tokens (including the encrypted keyword, the latest state of $w_{1}$, and the update count) and sends them to the server. Upon receiving the search tokens, the server sequentially retrieves all states of $w_{1}$ along with their corresponding encrypted entries and returns the results to the client. Finally, the client decrypts and filters the results.

### 4.3. Security Analysis

We now informally show that Veruna achieves forward privacy and **Type-II** backward privacy. Since the state is randomly generated, the value of each state that the server observes during an update is indistinguishable from a randomly drawn value, and the server cannot infer the future state relying on the current search token, which contains the current state and state token. Therefore, the forward privacy is guaranteed. For analyzing the backward privacy of Veruna, consider that during a search, the server computes a set of locations for keyword *w*, which was observed previously during updates. This information reveals the timestamp of each update for keyword *w*. Apart from this information, the server obtains nothing else. In particular, it cannot learn which delete operation corresponds to which add operation. Referring to the definition of the leakage function in Subsection III-D, after the above leakage is captured, the formal definition of Veruna’s leakage functions is as follows:

(9) $$\begin{array}{cc} \; & \;\;L^{Updt}\left(w,ind,op\right)=⊥,\\ \; & \;L^{Srch}\left(w\right)=\left(\mathrm{sp}\left(w\right),\mathrm{TimeDB}\left(w\right),\mathrm{Updates}\left(w\right)\right). \end{array}$$

According to Definition 2 of forward privacy and Definition 3 of backward privacy, our scheme achieves forward privacy and **Type-II** backward privacy.

Formally, Veruna’s adaptive security is stated in the following theorem.

**Theorem** **1.** *Assume that F is a secure pseudo-random function, while $H_{1}$, $H_{2}$ and $H_{3}$ are hash functions modeled as random oracles. Then, Veruna is an L-adaptively-secure SSE scheme with the leakage functions $L^{Updt}\left(w,ind,op\right)=⊥$ and $L^{Srch}\left(w\right)=\left(\mathrm{sp}\left(w\right),\mathrm{TimeDB}\left(w\right),\mathrm{Updates}\left(w\right)\right)$.*

**Proof.**  We use the $R_{\mathrm{EAL}}$-$I_{\mathrm{DEAL}}$ model defined in Subsection II-C to prove the security of Veruna. A sequence of games is constructed from ${R_{\mathrm{EAL}}}_{A}^{\Sigma }\left(\lambda \right)$ and reached to ${I_{\mathrm{DEAL}}}_{A,S}^{\Sigma }\left(\lambda \right)$. We prove that ${R_{\mathrm{EAL}}}_{A}^{\Sigma }\left(\lambda \right)$ and ${I_{\mathrm{DEAL}}}_{A,S}^{\Sigma }\left(\lambda \right)$ are indistinguishable by proving the indistinguishability between two adjacent games.

**Game**$\;\;G_{0}$: $G_{0}$ is the real-world game ${R_{\mathrm{EAL}}}_{A}^{\Sigma }\left(\lambda \right)$.

(10) $$\mathrm{Pr}\left({R_{\mathrm{EAL}}}_{A}^{\Sigma }\left(\lambda \right)=1\right)=\mathrm{Pr}\left(G_{0}=1\right).$$

**Game**$\;\;G_{1}$: The difference between $G_{1}$ and $G_{0}$ is that instead of using *F* to generate $k_{w}$, a random $k_{w}$ is chosen and stored in the mapping Key. For the subsequent query on *w*, the corresponding $k_{w}$ can be directly extracted from Key. Since we cannot distinguish the pseudo-random function *F* from the truly random function, $G_{1}$ and $G_{0}$ are indistinguishable.

**Game**$\;\;G_{2}$: The difference between $G_{2}$ and $G_{1}$ is that $G_{2}$ no longer calls $H_{1}$ to generate a location in the update protocol but uses random numbers instead. Concretely, it replaces $u\leftarrow H_{1}\left(k_{w},st_{c+1}\right)$ with $u\overset{\$}{\leftarrow }{\left\{0,1\right\}}^{\lambda }$ and executes $L[k_{w}||st_{c+1}]\leftarrow u$, where L is a mapping maintained by $G_{2}$. Then, $H_{1}[k_{w}||st_{c+1}]\leftarrow L[k_{w}||st_{c+1}]$ is executed in the search protocol, where $H_{1}$ is the table of the random oracles $H_{1}$. Thus, $H_{1}$ is not updated immediately, and when an adversary accesses $H_{1}[k_{w}||st_{c+1}]$ before a search query is issued, $H_{1}[k_{w}||st_{c+1}]$ will randomly generate a value $u^{*}$ that is not equal to *u*. If the adversary queries $H_{1}[k_{w}||st_{c+1}]$ again after the next search query, it will obtain the value *u* that has been updated to $H_{1}$. By observing the difference between the two queries, the adversary may know it is in-game $G_{2}$. Below, we show that the probability of this case is negligible.

This case will only occur if the adversary uses $k_{w}\left|\right|st_{c+1}$ to query $H_{1}$. Since $st_{c+1}$ is randomly generated, the adversary chooses $st_{c+1}$ with probability $\frac{1}{2^{\lambda }}+negl\left(\lambda \right)$. Assuming that a PPT adversary makes at most p=poly(λ) guesses, the probability of adversary chooses $st_{c+1}$ is $\frac{p}{2^{\lambda }}+p\cdot negl\left(\lambda \right)$. This probability is negligible and, therefore, $G_{2}$ and $G_{1}$ are indistinguishable, i.e.,

(11) $$\mathrm{Pr}\left(G_{1}=1\right)-\mathrm{Pr}\left(G_{2}=1\right)\leq \frac{p}{2^{\lambda }}+p\cdot negl\left(\lambda \right).$$

**Game**$\;\;G_{3}$: The difference between $G_{3}$ and $G_{2}$ is that in the update protocol of $G_{3}$, $H_{2}$ is processed in the same way as $H_{1}$ in $G_{2}$. Since the probability that the adversary guesses the correct key without knowing $k^{*}$ is $\frac{1}{2^{\lambda }}+negl\left(\lambda \right)$, and the probability that the adversary queries p=poly(λ) polynomially is $\frac{p}{2^{\lambda }}+p\cdot negl\left(\lambda \right)$, $G_{3}$ and $G_{2}$ are indistinguishable, i.e.,

(12) $$\mathrm{Pr}\left(G_{2}=1\right)-\mathrm{Pr}\left(G_{3}=1\right)\leq \frac{p}{2^{\lambda }}+p\cdot negl\left(\lambda \right).$$

**Game**$\;\;G_{4}$: The difference between $G_{4}$ and $G_{3}$ is that $H_{3}$ is modeled as a random oracle in the update protocol of $G_{4}$. Similar to the previous analysis, $G_{4}$ and $G_{3}$ are indistinguishable, i.e.,

(13) $$\mathrm{Pr}\left(G_{3}=1\right)-\mathrm{Pr}\left(G_{4}=1\right)\leq \frac{p}{2^{\lambda }}+p\cdot negl\left(\lambda \right).$$

**Game**$\;\;G_{5}$: The difference between $G_{5}$ and $G_{4}$ is that st in $G_{5}$ is generated on the fly during the search. Algorithm 2 shows the changes on the client side. $G_{5}$ uses Hist to record the update history since the last search and parse out the current update timestamp and set of files containing keyword *w*. Unlike $G_{4}$, $G_{5}$ randomly selects the query result of a random oracle without the information about st. Then, $st_{i}$ is generated when a search query is issued, and the random oracle is updated. From the adversary’s perspective, since $G_{4}$ and $G_{5}$ output three random strings in the update and $\left(k_{w},st_{c},c\right)$ in the search, $G_{4}$ and $G_{5}$ are completely indistinguishable, i.e.,

(14) $$\mathrm{Pr}\left(G_{4}=1\right)-\mathrm{Pr}\left(G_{5}=1\right)=0.$$

**Algorithm 2.** Game $G_{5}$$\underline{\mathrm{Setup}(\lambda )}$13: ifW[w]=⊥thenClient:14: $\;W\left[w\right]\overset{\$}{\leftarrow }{\left\{0,1\right\}}^{\lambda }$
1: L,E,C←emptymap15: endif
2: t←016: ParseHist[w]asTime[w]$\underline{\mathrm{Update}(k_{s},W,w,ind,op;T)}$17: ParseHist[w]asDB[w]Client:18: $\left(t_{0},t_{1},\cdots ,t_{c}\right)\leftarrow \mathrm{Time}\left[w\right]$
3: Add(t,op,ind)toHist[w]19: $st_{0}\leftarrow W\left[w\right]$
4: $L\left[t\right]\overset{\$}{\leftarrow }{\left\{0,1\right\}}^{\lambda }$20: c←|Time[w]|
5:$E\left[t\right]\overset{\$}{\leftarrow }{\left\{0,1\right\}}^{l+1}$21: fori=1tocdo
6: $C\left[t\right]\overset{\$}{\leftarrow }{\left\{0,1\right\}}^{\lambda }$22:$\;st_{i}\overset{\$}{\leftarrow }{\left\{0,1\right\}}^{\lambda }$
7: t←t+123:$\;H_{1}[k_{w}||st_{i}]\leftarrow L\left[t_{i}\right]$
8: Send(L,E,C)toserver24:$\;H_{3}[k_{w}||st_{i}]\leftarrow C\left[t_{i}\right]⊕st_{i-1}$$\underline{\mathrm{Search}(k_{s},W,w;T)}$25: endforClient:26: forind∈DB[w]do
9: ifKey[w]=⊥then27: $\;H_{2}[k_{w}^{*}||st_{i}]\leftarrow E\left[t_{i}\right]$
10: $\;\mathrm{Key}\left[w\right]\overset{\$}{\leftarrow }{\left\{0,1\right\}}^{\lambda }$⊕(ind||op)
11: endif28: endfor
12: $k_{w}\left|\right|k_{w}^{*}\leftarrow \mathrm{Key}\left[w\right]$29: $\mathrm{Send}\;(k_{w},st_{c},c)\;\mathrm{to}\;\mathrm{server}$
**Simulator**: In ${I_{\mathrm{DEAL}}}_{A,S}^{\Sigma }\left(\lambda \right)$, the simulator S generates a view according to the given leakage function. Algorithm 3 shows simulator S, which maintains three maps for random Oracle queries and a counter for updates. The value of each map is also randomly generated during the update. Unlike $G_{5}$, S uses $\underline{w}\leftarrow \min\mathrm{sp}\left(w\right)$ to represent the timestamp of the first search keyword *w* in the search, and the leakage function TimeDB[w] and Updates[w] are used directly as input to parse the timestamp of each update and the set of files containing the keyword currently instead of counting against the update history Hist. Then, S randomly generates $st_{i}$ and updates the random oracle based on the above information. The view generated by the S is completely indistinguishable from $G_{5}$, and hence

(15) $$\mathrm{Pr}\left({I_{\mathrm{DEAL}}}_{A,S}^{\Sigma }\left(\lambda \right)=1\right)-\mathrm{Pr}\left(G_{5}=1\right)=0.$$

**Algorithm 3.** Simulator S$\underline{S.\mathrm{Setup}()}$13: ifW[w]=⊥thenClient:14: $\;W\left[w\right]\overset{\$}{\leftarrow }{\left\{0,1\right\}}^{\lambda }$
1: L,E,C←emptymap15: endif
2: t←016: ParseTimeDB[w]asDB[w]$\underline{S.\mathrm{Update}()}$17: $\left(t_{0},t_{1},\cdots ,t_{c}\right)\leftarrow \mathrm{Updates}\left[w\right]$Client:18: $st_{0}\leftarrow W\left[\underline{w}\right]$
3: $L\left[t\right]\overset{\$}{\leftarrow }{\left\{0,1\right\}}^{\lambda }$19: c←|Updates[w]|
4: $E\left[t\right]\overset{\$}{\leftarrow }{\left\{0,1\right\}}^{l+1}$20: fori=1tocdo
5: $C\left[t\right]\overset{\$}{\leftarrow }{\left\{0,1\right\}}^{\lambda }$21: $\;st_{i}\overset{\$}{\leftarrow }{\left\{0,1\right\}}^{\lambda }$
6: t←t+122: $\;H_{1}[k_{w}||st_{i}]\leftarrow L\left[t_{i}\right]$
7: Send(L,E,C)toserver23: $\;H_{3}[k_{w}||st_{i}]\leftarrow C\left[t_{i}\right]⊕st_{i-1}$$\underline{S.\mathrm{Search}(L^{Srch}\left(w\right))}$24: endforClient:25: forind∈DB[w]do
8: $\underline{w}\leftarrow \min\mathrm{sp}\left(w\right)$26: $\;H_{2}[k_{w}^{*}||st_{i}]\leftarrow E\left[t_{i}\right]$
9: ifKey[w]=⊥then⊕(ind||op)
10: $\;\mathrm{Key}\left[w\right]\overset{\$}{\leftarrow }{\left\{0,1\right\}}^{\lambda }$27: endfor
11: endif28: $\mathrm{Send}\;(k_{w},st_{c},c)\;\mathrm{to}\;\mathrm{server}$
12: $k_{w}\left|\right|k_{w}^{*}\leftarrow \mathrm{Key}\left[w\right]$

Finally, utilizing (10) to (15) leads to

(16) $$\mathrm{Pr}\left({R_{\mathrm{EAL}}}_{A}^{\Sigma }\left(\lambda \right)=1\right)-\mathrm{Pr}\left({I_{\mathrm{DEAL}}}_{A,S}^{\Sigma }\left(\lambda \right)=1\right)\leq negl\left(\lambda \right).$$

 □

### 4.4. Efficiency of Veruna

As the number of operations for the client and server is constant during the update, the computational complexity of both the client and server is O(1) in the update operations. The communication complexity of updating a single identifier-keyword pair is also O(1). For search operations, the client requires $a_{w}$ hash operations and XOR operations, while the server requires $a_{w}$XOR operations and $2a_{w}$ hash operations, where $a_{w}$ is the total number of updates for keyword *w*. Therefore, the computational complexity of the search is $O(a_{w})$ for both the client and server; the same is true for the communication. The client stores a λ bit secret key $k_{s}$ and a map W containing all keywords’ states. The size of W is O(|W|), where |W| is the total number of keywords. After *N* pairs of values are added to the map T, the storage at the server is O(N).

For FSSE [17], the server-side index size is N·(λ+1+l+2δ), where *N* is the number of entries, λ is the length of the key, *l* is the identifier’s size of files, 1 bit is the size of the operation type, and δ is the identifier’s size of blocks. By contrast, for Veruna, the server-side index size is N·(λ+1+l), where λ is the length of the state token. Compared to FSSE, Veruna does not need to store additional block identifiers, which reduces the storage overhead.

Moreover, Veruna is a fast scheme. Table 3 compares the computation and communication cost of our scheme with Fsse [17], Mitra [24], and Qos [26] on the client and server during the search and update process as well as the security of the scheme. In Table 2, $\tilde{O}$ hides polylog factors, $a_{w}$ denotes the number of updates for keyword *w*, $n_{w}$ denotes the current matching count for *w*, $i_{w}$ denotes the number of additions for *w*, *N* is the sum of update counts, and |W| is the sum of different keyword counts. It can be observed that Veruna’s computation and communication costs are superior to QoS’s. Compared with Fsse, the main distinction between Veruna and Fsse lies in the communication cost of Veruna’s server during the search, which is higher than that of Fsse. Specifically, the communication cost at the server side for Fsse is $O(n_{w})$, whereas for Veruna, it is $O(a_{w})$. This is because Veruna’s server sends all update entries matching keyword *w* to the client, while Fsse’s server sends file identifiers containing *w* to the client. However, Fsse lacks consideration for backward privacy. Veruna and Mitra both belong to Type-II backward privacy schemes. However, the computation and communication costs of Veruna’s client are in constant order during the search, whereas for Mitra, they are related to $a_{w}$. In Section 5, we implemented Veruna and compared it with Mitra and Qos on real datasets. The experimental results show that Veruna’s search performance outperforms Mitra and QoS. Note that Veruna has only one cryptographic operation (compute the state of the keyword) in the server, which improves server performance and deploys more easily.

### 4.5. Veruna with Cleanup

In Veruna of Algorithm 1, the size of the encrypted database grows as the client continues to execute update operations. Moreover, the server will return the deleted file identifier to the client for each search query. For example, the server repeatedly returns the deleted entries to the client for the same query, which undoubtedly affects the client’s communication cost and workload. To this end, we design a modified version of Veruna with a ‘clean-up’ operation [14,24,26]. The server performs a clean-up operation during the search while the client re-encrypts the remaining entries and sends them to the server. The blue box in Algorithm 2 shows the modifications made to Veruna of Algorithm 1.

The overall structure of Algorithm 4 is the same as that of Algorithm 1. However, the server in Algorithm 4 cleans up all the retrieved entries during the search (line 29). After that, the client executes a re-encryption operation (lines 42–46) on the elements in Res. It is worth noting that since Veruna uses deterministic encryption, re-encrypting the same element will result in the same ciphertext, and the server can use this information to obtain which of the previously observed entries disappeared, which will break the privacy of the scheme. To solve this problem, we introduce a counter map Cnt, which will grow after every search (line 42). Letting Cnt[w] as the input of the encryption operation can effectively ensure the security of the scheme (lines 12–14). In terms of security, Algorithm 4 leaks no information in the update operation, and the server can only obtain the updated timestamp in the search operation. Thus, this construction has the same forward and backward privacy as Veruna of Algorithm 1. Algorithm 4, however, avoids repeated operations related to the deleted entries in each search query and periodically cleans up the entries stored in the server. Without new updates, this construction’s computational and communication complexity in the search are both $O(n_{w})$. At the same time, the storage size of the server is $O(N^{*})$, where $n_{w}$ is the number of current files matching the keyword *w* and $N^{*}$ is the number of remaining entries in the server.
**Algorithm 4.** Modified Veruna with cleanup$\underline{\mathrm{Setup}(\lambda )}$15: $\mathrm{Send}\;(u_{c+1},e_{c+1},C_{st_{c}})\;\mathrm{to}\;\mathrm{server}$30: endforClient:Server:31: SendValtoclient1: $k_{s}\overset{\$}{\leftarrow }{\left\{0,1\right\}}^{\lambda }$16: $T\left[u_{c+1}\right]\;=\;\left(e_{c+1},C_{st_{c}}\right)$Client:2: W←emptymap$\underline{\mathrm{Search}(k_{s},W,\mathrm{Cnt},w;T)}$32: Res←∅3: $\begin{array}{c} \mathrm{Cnt}\leftarrow 0 \end{array}$Client:33: fori=1to|Val.size|doServer:17: $k_{w}\;\left|\right|\;k_{w}^{*}\leftarrow F(k_{s},w)$34: $\;(ind_{i}||op_{i})\leftarrow \mathrm{Val}\left[i\right]⊕H_{2}(k_{w}\left||\mathrm{Cnt}\left[w\right],i\right)$4: T←emptymap18: $\left(st_{c},c\right)\leftarrow W\left[w\right]$35: $\;\mathrm{if}\;op_{i}\;=\;add\;\mathrm{then}$$\underline{\mathrm{Update}(k_{s},W,\mathrm{Cnt},w,ind,op;T)}$19: $\mathrm{if}\;(st_{c},c)\;=\;⊥\mathrm{then}$36: $\;\;\mathrm{Res}\leftarrow \mathrm{Res}\cup ind_{i}$Client:20: return∅37: else5: $k_{w}\;\left|\right|\;k_{w}^{*}\leftarrow F(k_{s},w)$21: endif38: $\;\;\mathrm{Res}\leftarrow \mathrm{Res}\setminus ind_{i}$6: $\left(st_{c},c\right)\leftarrow W\left[w\right]$22: $\mathrm{Send}\;(k_{w},st_{c},c,\mathrm{Cnt}\left[w\right])\;\mathrm{to}\;\mathrm{server}$39: endif7: $\mathrm{if}\;(st_{c},c)\;=\;⊥\mathrm{then}$Server:40: endfor8: $\;{st}_{0}\overset{\$}{\leftarrow }{\left\{0,1\right\}}^{\lambda }\;,\;c\leftarrow 0$23: Val←∅41: ReturnRes9: endif24: fori=cto1do42: $\begin{array}{c} \mathrm{Cnt}\left[w\right]\leftarrow \mathrm{Cnt}\left[w\right]+1 \end{array}$10: ${st}_{c+1}\overset{\$}{\leftarrow }{\left\{0,1\right\}}^{\lambda }$25: $\;u_{i}\leftarrow H_{1}(k_{w}||\mathrm{Cnt}\left[w\right],st_{i})$43: $\begin{array}{c} W\left[w\right]\leftarrow ⊥ \end{array}$11: $W\left[w\right]\leftarrow \left(st_{c+1},c+1\right)$26: $\;\left(e_{i},C_{st_{i-1}}\right)\leftarrow T\left[u_{i}\right]$44: $\begin{array}{c} \mathrm{for}\;ind\;\in \;\mathrm{Res}\;\mathrm{do} \end{array}$≪Inparallel12: $\begin{array}{c} u_{c+1}\leftarrow H_{1}(k_{w}||\mathrm{Cnt}\left[w\right],st_{c+1}) \end{array}$27: $\;\mathrm{Val}\left[i\right]\leftarrow e_{i}$45: $\;\begin{array}{c} Run\;\mathrm{Update} \end{array}$13: $\begin{array}{c} e_{c+1}\leftarrow \left(ind||op\right)⊕H_{2}(k_{w}^{*}\left||\mathrm{Cnt}\left[w\right],c+1\right) \end{array}$28: $\;st_{i-1}\leftarrow C_{st_{i-1}}⊕H_{3}(k_{w}||\mathrm{Cnt}\left[w\right],st_{c+1})$46: $\begin{array}{c} \mathrm{end}\;\mathrm{for} \end{array}$14: $\begin{array}{c} C_{st_{c}}\leftarrow st_{c}⊕H_{3}(k_{w}||\mathrm{Cnt}\left[w\right],st_{c+1}) \end{array}$29: $\;\begin{array}{c} delete\;T[u_{i}] \end{array}$


## 5. Performance Evaluation

In this section, we compare Veruna with the existing state-of-the-art **Type-II** scheme Mitra [24] and **Type-III** scheme QoS [26]. To the best of our knowledge, considering both update and search efficiency comprehensively, Mitra and QoS are the most efficient **Type-II** and **Type-III** schemes, respectively. We use the publicly available codes for the Mitra and QoS schemes in our evaluation comparison. Additionally, to minimize the impact of parameters on experimental results, we maintain the same operating environment and adopt similar function settings as much as possible. Since we have analyzed the storage overhead of Veruna in Section 4.4, the evaluation metrics used in this section only include the time cost of searches and updates and the effect of deletions.

### 5.1. Implementation and Settings

We implement Veruna in Python and use the PyCrypto library to achieve symmetric cryptographic operations. Specifically, we use AES-256 to realize PRF *F* and all hash operations achieved by SHA-256. We test the performance of the schemes compared using the data from Enron email dataset (Enron Email Dataset: available online at https://www.cs.cmu.edu/~enron/, accessed on 21 October 2009), which is derived from the real world and consists of multiple folders containing email messages from about 150 different users. We choose 30,109 emails in the sent-email folder as the file set and apply the keyword extraction process of [57,58] to obtain 77,000 unique keywords, which exclude some stopwords like ‘a’, ‘the’, and ‘so’. Finally, we obtain variable datasets with size $\left|\mathrm{DB}|=2\times (10^{2}∼10^{6}\right)$. For each dataset, we also choose keywords that have $3\times (10^{0}∼10^{4})$ matching documents. All experiments were conducted on workstations equipped with an Intel(R) Core(TM) i7-14700K 3.40 GHz CPU, 32 GB and 16 GB RAM, running Windows 11 (64-bit). All the experiments are repeated 10 times, and the results are averaged over the 10 runs.

### 5.2. Search and Update Performance

**Figure 5:**
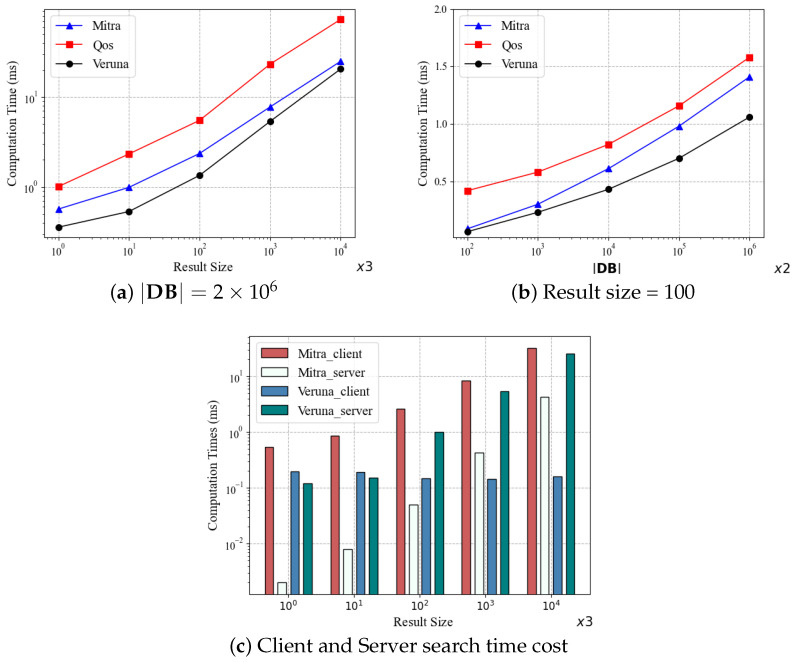
Search time comparison of Mitra, Qos and Veruna.

In Figure 5, we compare the execution times of Mitra, Qos, and Veruna in the search operation. Specifically, Figure 5a shows the execution time of each scheme for searching different result sizes when the database size is $\left|\mathrm{DB}\right|=2\times 10^{6}$. As expected, the execution times of all the schemes increase with the result size. It can be seen that Veruna outperforms Mitra and Qos. For example, at the result size of $10^{3}$, Veruna is four times faster than Qos and 1.5 times faster than Mitra. It is worth noting that Mitra is an extremely fast scheme, which is 145 to 253 times faster than Fides [22]. But our Veruna is even faster than Mitra. Figure 5b depicts the execution times of the three schemes as the functions of the database size given the result size of 100. It can be seen that the execution time increases almost linearly with the database size. This should be compared with Figure 5a, which shows that the influence of the result size on the execution time is much stronger. It can be seen again that Veruna outperforms Mitra and Qos. According to the above experiments, the performance of **Type-III** scheme Qos is worse than Mitra and Veruna. QoS is not good at executing searches with small deletion rates (10% in our experiments). In Section 5.3, we will investigate the effect of different deletion rates on the search performance.

To further investigate the search performance of Mitra and Veruna, which are both **Type-II** schemes, we count the time consumption of both schemes on the client and server, respectively, as shown in Figure 5c. On the client side, as the result size increases from $3\times 10^{0}$ to $3\times 10^{4}$, Mitra’s time cost rises from 0.54 ms to 32.4 ms, while Veruna consistently remains around 0.16 ms. The cost of Veruna is almost independent of the result size, which can be explained by the computing steps required by the client. Specifically, Mitra needs to generate a list containing all the file locations that match the keyword, and the list size increases with the result size. By contrast, Veruna only needs to obtain encrypted keywords and the keyword state as search tokens. Therefore, the cost of Veruna is lower and independent of the result size. On the server side, Veruna’s cost is higher than Mitra’s because Veruna has additional location computation and keyword state backtracking operations. But this does not alter the fact that Veruna outperforms Mitra in terms of total time cost, as demonstrated in Figure 5a.

**Figure 6:**
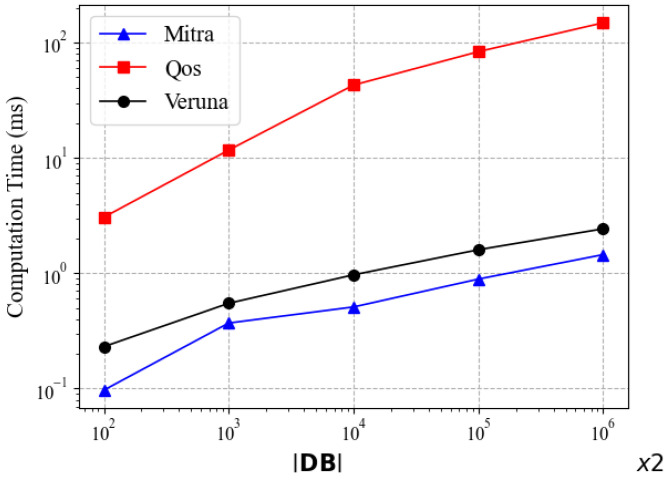
Update time comparison of Mitra, Qos and Veruna.

Figure 6 shows the update computation times of Mitra, Qos, and Veruna as the functions of the database size. It can be seen that the time costs of Veruna and Mitra are dramatically lower than that of Qos. Veruna’s performance is slightly worse than Mitra’s. For example, at the database size of $2\times 10^{3}$, Veruna takes about 0.5 ms and Mitra 0.4 ms. This can be explained by the update steps required. Compared to Mitra, Veruna requires additional computation for the state of the keyword as well as the state token, which leads to an increase in the update time cost. Although these additional calculations in the update are not insignificant, they enable Veruna to perform better during the search, as demonstrated in the above-mentioned experiments of search performance.

### 5.3. Effect of Deletions

In all the above experiments, we set the deletion rate of entries to 10%, and in this case, Qos performs particularly poorly. We further experiment to investigate the impact of different deletion rates on the search time of each scheme. In this experiment, we fix the database size to $\left|\mathrm{DB}\right|=2\times 10^{6}$ and consider two cases: (a) small result size of 100 and (b) large result size of $2\times 10^{4}$. Given a result size, we set the deletion rate between 0∼70%. With the result size 100, for example, a 10% deletion rate means we inserted 111 entries and deleted 11.

**Figure 7:**
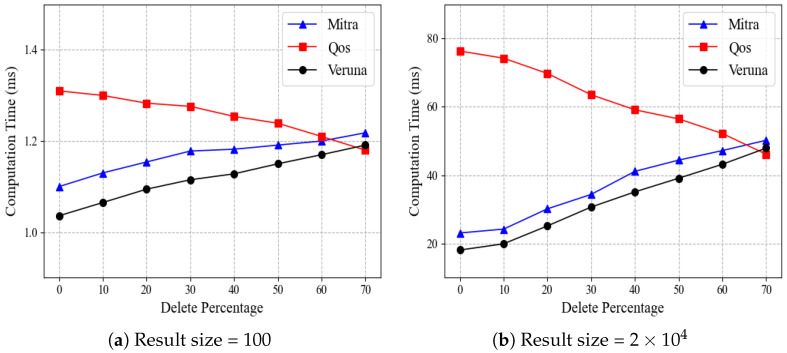
Effect of deletions on search time.

Figure 7 compares the search performance of Mitra, Qos, and Veruna by varying the deletion rate, where Figure 7a,b show the results for the small result size and large result size, respectively. As the deletion rate increases, the time costs of Mitra and Veruna grow progressively, while the opposite is true for Qos. Specifically, for result size $=2\times 10^{4}$, when the deletion rate increases from 50% to 60%, Mitra’s time cost rises from 41.1 ms to 44.4 ms, Veruna’s from 35.2 ms to 39.1 ms, while Qos’s decreases from 59.2 ms to 56.5 ms. This trend is slight for the result size of 100 and more significant for the result size of $2\times 10^{4}$. The results of Figure 7 are to be expected because the search operation of Mitra and Veruna is related to $a_{w}$, where $a_{w}$ is the total number of updates for keyword *w*. At the same time, QoS is a quasi-optimal scheme related to $n_{w}$, where $n_{w}$ is the number of results currently matching *w*. As the deletion rate increases, the total number of updates for keyword *w* also increases, while the number of files matching *w* decreases. In addition, when the deletion rate is lower than 60, the time cost of Qos is higher than those of Mitra and Veruna, and Veruna has the lowest time cost. Note that in practice, many deletions do not happen frequently. Instead, a moderate number of deletions is the norm.

**Note on Applicability to IoT Devices:** Although the comparison experiments were conducted on a high-performance workstation, the Veruna scheme is designed to be adaptable for use in IoT environments with limited computational capabilities. In such scenarios, IoT devices perform lightweight operations such as data collection and initial encryption. The more demanding processes, such as handling search queries and updating encrypted indices, can be offloaded to cloud servers with greater computational power. This division of tasks ensures that the scheme remains practical for IoT use cases without compromising overall performance or security.

## 6. Conclusions and Future Work

In this work, we proposed Veruna, which is a DSSE scheme for cloud-assisted IIoT with fast search performance and strong security. To ensure that Veruna holds the forward and (for Type-II) backward privacy, we have used the state token to connect the state of each keyword and construct a state chain structure to support it, which only requires a little server storage. Moreover, unlike most existing backward privacy schemes, which introduce additional complex cryptographic operations and increase the server’s workload, Veruna only needs to perform a simple XOR state backtracking on the server without relying on other complex operations. This advantage is desirable for practical deployment in cloud-assisted IIoT. Finally, we compared Veruna with two state-of-the-art schemes in experiments, and the results demonstrated that our scheme was more effective.

Regarding future work, we refer to some of the latest research results and discover widespread interest in the expressiveness of DSSE and non-interactive search. More research is needed on implementing non-interactive range keyword queries and verification functions in DSSE, and we aim to pursue research in this direction. Implementing these features will facilitate the practical deployment of DSSE in various IoT environments.

## Figures and Tables

**Table 1:** Comparison of existing schemes with the proposed Veruna.

Scheme	Computation		Communication			Backward
	Search	Update	Search	RT	Update	Privacy
Sophos [14]	$O(a_{w})$	O(1)	$O(n_{w})$	1	O(1)	None
Fsse [17]	$O(a_{w})$	O(1)	$O(n_{w})$	1	O(1)	None
Moneta [22]	$\tilde{O}\left(a_{w}\log N+\log^{3}N\right)$	$\tilde{O}\left(\log^{2}N\right)$	$\tilde{O}\left(a_{w}\log N+\log^{3}N\right)$	3	$\tilde{O}\left(\log^{3}N\right)$	I
Diana [22]	$O(a_{w})$	$O(\log a_{w})$	$O(d_{w}\log a_{w}+n_{w})$	2	O(1)	III
Janus [22]	$O(n_{w}d_{w})$	O(1)	$O(n_{w})$	1	O(1)	III
Janus++ [23]	$O(n_{w}d_{w})$	O(1)	$O(n_{w})$	1	O(1)	III
Mitra [24]	$O(a_{w})$	O(1)	$O(a_{w})$	2	O(1)	II
Orion [24]	$O(n_{w}\log^{2}N)$	$O(\log^{2}N)$	$O(n_{w}\log^{2}N)$	O(logN)	$O(\log^{2}N)$	I
Hour [24]	$O(n_{w}\log d_{w}\log N)$	$O(\log^{2}N)$	$O(n_{w}\log d_{w}\log N)$	$O(\log d_{w})$	$O(\log^{2}N)$	III
${\mathrm{SD}}_{a}$ [26]	$O(a_{w}+\log N)$	O(logN)	$O(a_{w}+\log N)$	2	O(logN)	II
Qos [26]	$O(n_{w}\log i_{w}+\log^{2}|W|)$	$O(\log^{3}N)$	$O(n_{w}\log i_{w}+\log^{2}|W|)$	O(log|W|)	$O(\log^{3}N)$	III
Veruna	$O(a_{w})$	O(1)	$O(a_{w})$	2	O(1)	II

*N* is the number of keyword/document pairs, |W| is the number of total keywords, and RT is the number of roundtrips in the search protocol. For keyword w∈W, $a_{w}$ is the total number of keyword updates, $d_{w}$ is the number of deleted operations for *w*, $i_{w}$ is the number of add operations for *w*, and $n_{w}$ is the number of results currently matching *w*. The notion $\tilde{O}$ hides polylog factors, and hence $\tilde{O}\left(A\right)>O\left(A\right)$.

**Table 2:** Major functions of our scheme.

Update keyword/identifier pair $\left(w_{1},ind_{1}\right),\left(w_{1},ind_{2}\right),\left(w1,ind_{3}\right)$
**Client:**
(1) create the encrypted inverted index
(2) send the encrypted indices and file to the server
**Server:**
(3) add received messages to the encrypted database (EDB)
Keyword search $w_{1}$
**Client:**
(1) create a search token for $w_{1}$
(2) send the search token to the server
**Server:**
(3) search the state of $w_{1}$ along with encrypted entries from the invert index
(4) return the results to the client
**Client:**
(5) decrypt and filter the results

**Table 3:** Comparison of existing schemes with proposed Veruna.

Scheme	Computation				Communication			Forward Privacy	Backward Privacy
	Search		Update		Search		Update		
	Client	Server	Client	Server	Client	Server			
Fsse	O(1)	$O(a_{w})$	O(1)	O(1)	O(1)	$O(n_{w})$	O(1)	✓	×
Mitra	$O(a_{w})$	$O(a_{w})$	O(1)	O(1)	$O(a_{w})$	$O(a_{w})$	O(1)	✓	II
Qos	$O(\log^{2}|W|)$	$O(n_{w}\log i_{w})$	$O(\log^{3}N)$	O(1)	$O(\log^{2}|W|)$	$O(n_{w}\log i_{w})$	$O(\log^{3}N)$	✓	III
Veruna	O(1)	$O(a_{w})$	O(1)	O(1)	O(1)	$O(a_{w})$	O(1)	✓	II

## Data Availability

Data are contained within the article.
